# The Development of the Ascending Aortic Wall in Tricuspid and Bicuspid Aortic Valve: A Process from Maturation to Degeneration

**DOI:** 10.3390/jcm9040908

**Published:** 2020-03-26

**Authors:** Nimrat Grewal, Adriana C. Gittenberger-de Groot, Jan von der Thusen, Lambertus J. Wisse, Margot M. Bartelings, Marco C. DeRuiter, Robert J.M. Klautz, Robert E. Poelmann

**Affiliations:** 1 Department of Cardiothoracic Surgery, Leiden University Medical Center, 2300RC Leiden, The Netherlands; R.J.M.Klautz@lumc.nl; 2 Department of Anatomy and Embryology, Leiden University Medical Center, 2300RC Leiden, The Netherlands; acgitten@lumc.nl (A.C.G.-d.G.); l.j.wisse@lumc.nl (L.J.W.); m.m.bartelings@lumc.nl (M.M.B.); m.c.de_ruiter@lumc.nl (M.C.D.); 3 Department of Cardiology, Leiden University Medical Center, 2300RC Leiden, The Netherlands; R.E.Poelmann@lumc.nl; 4 Department of Pathology, Erasmus Medical Center, 3015GD Rotterdam, The Netherlands; j.vonderthusen@erasmusmc.nl; 5 Institute of Biology, Animal Sciences and Health, Leiden University, 2300RC Leiden, The Netherlands

**Keywords:** bicuspid aortic valve, tricuspid aortic valve, development, aortopathy, histopathology

## Abstract

Background: Patients with a bicuspid aortic valve (BAV) have an increased risk for aortic dilation and dissection. In this study, we provide a histological stratification of the developing aorta in the tricuspid aortic valve (TAV) and the BAV populations as a reference for future studies on aortopathy and related syndromes. Methods: Non-dilated TAV and BAV ascending aortic wall samples were collected, including 60 TAV (embryonic–70 years) and 32 BAV specimens (fetal–72 years, categorized in eight age groups. Results: In TAV, intimal development starts in the neonatal phase. After birth, the thickness of the medial layer increases significantly by increase of elastic lamellae up to and including the “young child” phase stabilizing afterwards. The BAV shows already prenatal intimal thickening becoming significantly thinner after birth subsequently stabilizing. In BAV, increase in elastic lamellae is seen between the young child and the adolescent phases, stabilizing afterwards. Conclusions: Vascular development in TAV is described in three phases: maturation, stabilization, and degeneration. For BAV, the development can be described in two phases: maturation (already prenatally) and degeneration. After birth, the development of the aorta is characterized by degeneration, leading to weakening of the ascending aortic wall and increasing the risk of aortopathy.

## 1. Introduction

The vascular system within the thorax contains major elastic arteries, including the ascending and the descending aorta, the pulmonary trunk, and the pulmonary arteries. Development of the vascular system involves a complex sequence of inductive and differentiating signals that lead to vasculogenesis starting by the formation of an endothelial plexus in the splanchnic mesoderm. The differentiation of the great arteries starts by stabilization of the endothelial network (arteriogenesis) with the recruitment of vascular smooth muscle cells (VSMCs) from the surrounding splanchnic mesoderm as well as the neural crest and through endothelial smooth muscle cell transformation [1]. Throughout the vascular system, there are many sources for these VSMCs [2,3,4]. In general, the great arteries acquire an elastic phenotype during development opposed to a muscular phenotype of the more peripheral arteries. The elastic arteries have specific characteristics that differ between the ascending and the descending aorta [5] as well as the pulmonary trunk [6].

The morphogenetic processes underlying the differences in the structure of the elastic arteries [5] and the muscular arteries are still poorly understood. Most probably, these are the results of origin of the endothelial and the VSMC populations in combination with the role of hemodynamics [7]. The great arteries in general are more influenced by high shear stress and pressure.

Developmental data on the origin of the endothelial and the VSMCs of the ascending aorta show a prominent role for neural crest derived cells as well as second heart field derived cells [8,9]. It is important to realize that these cell types are not only present in the ascending aortic wall but are also part of the forming semilunar valves of both the aorta and the pulmonary trunk, although in a different composition [10]. Therefore, an early embryonic defect of the neural crest cells and/or the second heart field cells could lead to the development of both an abnormal valve and defects in the VSMC differentiation, as is seen in patients with a bicuspid aortic valve (BAV) [11]. 

In the adult vascular wall, the VSMCs are highly specialized cells with contraction and regulation of blood vessel tone-diameter as primary function. Unlike cardiac muscle cells, adult VSMCs are not terminally differentiated and retain the possibility to undergo a phenotypic switch in response to local environmental cues [12]. During vascular development, VSMCs play a key role in differentiation of the vessel wall and exhibit high rates of proliferation and production of extracellular matrix components (e.g., collagen, elastin, fibrillin, and proteoglycans) while acquiring contractile properties [13]. At this stage of development, VSMCs form numerous gap junctions with endothelial cells, and this process of investment of endothelial tubes with VSMCs is crucial for vascular maturation [14]. In adult human blood vessels, the VSMCs are characterized by a low rate of proliferation/turnover and a very low rate of synthesis of extracellular matrix components and thus are completely dedicated to carry out their contractile function. However, in response to vascular injury, the VSMCs can dramatically increase their rate of proliferation, migration, and synthetic capacity, which plays a crucial role in vascular repair [13,15]. The ability to switch between a “contractile” and a “synthetic” phenotype is an important property of the differentiated VSMCs. However, the possibility to undergo a phenotypic switch in response to environmental cues also plays an important role in the development of major diseases, such as atherosclerosis [15,16,17]. 

In our previous research, we concluded that, in patients with a developmental defect in aortic valvulogenesis, being a BAV, the ascending aortic wall is characterized by VSMC immaturity due to a lack of differentiation of the VSMCs. While comparing nondilated and dilated tricuspid aortic valve (TAV) and BAV specimens, we further found that the adult intimal layer of the latter was significantly thinner compared to the TAV specimens [18,19]. In the early 1950s, Wilens described the intima of the aorta at birth consisting merely of a single layer of endothelium superimposed directly on its own reticular basement membrane or on that of the innermost elastic lamella of the media [20]. The infantile intima is merely a surface layer of endothelial cells. Postnatally, the aortic intima acquires a subendothelial fibrous zone of rather uniform width and becomes separated from the media by a zone of randomly arrayed VSMCs and elastic tissue [20,21]. Based on the abovementioned historical description of the intimal layer, the BAV intima seems premature. As, to this day, only adult ascending aortic wall specimens have been studied—and these have mostly been studies about atherosclerosis—we cannot decide whether the ascending aortic wall features in BAV are due to a delay in maturation or due to an underlying maturation defect. 

Understanding the development of the ascending aorta ranging from fetal stage to adulthood is mandatory to generate an overall view of the aging aorta as a reference to compare with syndromes associated with aortopathy such as BAV. This triggered the current study in which we investigated an upclimbing series of aortic wall specimens in the normally developed TAV from early to late fetal through neonatal, child, and adolescent stages to the already earlier investigated older adult population [18,19]. These specimens were then compared to age matched aortic wall tissues of BAV patients.

The aim of this study is to generate an overview of the developing aorta in the TAV and the BAV populations as a reference for future studies on aorta histopathology and aortopathy. 

## 2. Material and Methods

### 2.1. Patients and Tissue Samples and Ethical Approval 

All adult samples included in this study were non-dilated, defined as a diameter of the ascending aorta of less than 45 mm. Serial sections of postmortem fetal hearts were selected from the Leiden Collection (Department of Anatomy and Embryology, LUMC, Leiden, the Netherlands) in order to examine the ascending aortic wall of BAV and TAV. The age of these fetal specimens ranged from 10 weeks + days gestational age (GA) to 15 years. Adolescent BAV and TAV and adult TAV specimens were obtained post-mortem from the Pathology Department (LUMC). Additionally, young child to adolescent TAV specimens (age ranging from 2–18 years) were obtained from the Pathology Department of the Erasmus Medical Center (EMC) in Rotterdam. The purpose of collecting these valuable samples in the EMC was to use them as a “reference group” to compare to aortopathy samples. Adult BAV and TAV specimens were obtained during surgery from the aortotomy site when the preferred stentless aortic root replacement was performed [17] in the LUMC. We only included patients with an isolated BAV, while patients with a congenital cardiac anomaly besides a BAV were excluded (e.g., coarctation of the aorta, ventricular septal defect, Tetralogy of Fallot). None of the included patients died of a cardiac or a vessel-related cause. A total of 60 TAV (age ranging from 10 weeks GA–70 years) and 32 BAV (age ranging from 17.5 weeks + days GA–72 years) patients were included (Table 1). 

This study was undertaken in accordance with the local ethics committee and the Dutch regulation for the proper use of human tissue for medical research purposes. 

Embryonal, fetal, neonate, and infant aortic specimens used in our manuscript were obtained from the Leiden Collection of (malformed) hearts obtained from autopsies (Department of Anatomy and Embryology, Leiden, The Netherlands), a collection of hearts preserved in ethanol and glycerine dating from the 1950s in an era where no formal approval was in effect. The material was anonymized, and no privacy rules were violated. Furthermore, this study was conducted in accordance with the LUMC institutional guidelines for the use of human tissue and the Declaration of Helsinki. 

The World Health Organization suggested the following age categories in 2007 on the basis of earlier described pediatric categories [22]: premature < 38 weeks gestational age; neonate 0 < 30 days of age; infant 1 month < 2 years; young child 2 < 6 years; child 6 < 12 years, adolescent 12 < 18 years, young adults 18 < 21years and adults > 21 years of age. According to their definition, we clustered our material in 16 categories (Table 1). As the “child” group only contained one patient of 6 years in the TAV group, we decided to combine the young child and the child groups, which are collectively called the “young child” group hereafter. 

### 2.2. Sample Processing, Routine Histology, and Immunohistochemistry

The sectioning and staining protocols were described previously [23]. In summary, specimens obtained from hearts from our collection in the LUMC were fixed in formalin, decalcified, embedded in paraffin, and subsequently sectioned (4 μm). The sections were stained with hematoxylin-eosin (HE), resorcin fuchsin (RF), and Movat pentachrome. The immunohistochemical staining protocol used for αSMA (1/5000 -A2547, Sigma-Aldrich Chemie, Zwijndrecht, the Netherlands) was described in our previous study [18]. All specimens were evaluated by two independent, experienced histopathologists who were blinded to the clinical data. 

To describe the aortic wall in a standardized way, we used terms from the grading system described in the recent aortic consensus paper [24]. Terms used in this scoring system are overall medial degeneration (EMD), elastic fiber fragmentation and loss (EFF/L), elastic fiber thinning (EFT), elastic fiber disorganization (EFD), mucoid extra cellular matrix accumulation (MEMA), and smooth muscle cell nuclei loss (SMCNL). In our previous study [25], we suggested a modification of the aortic consensus statement grading system: the so called pathology score with addition of the intimal thickness and a number of immunohistochemical based features: (1) medial VSMC differentiation; (2) expression of αSMA; (3) intimal architecture including subendothelial layer structure and the absolute intimal thickness in μm; (4) intimal atherosclerosis indexed as 0 (none), 2 (mild), 4 (moderate), or 6 (severe). Most of the features scored were, however, related to cardiovascular pathology and therefore were not applicable to the pre- and the postnatal normal aortic tissues. In the current study, we performed a part of the pathology scoring system to describe the appearance of immaturity. We focused on the development of the elastic lamellae and the intimal layer with aspects of EFT, EFF, and MEMA. In RF stained sections, the maximum intimal and medial thicknesses were quantified in μm. We further calculated the ratio between the vessel’s intima thickness and the total vessel wall thickness and counted the number of elastic lamellae in the media and the interlamellar distance in the inner, the middle, and the outer media.

### 2.3. Histologic Parameters and Morphometry

Sections were studied with a Leica BM500 microscope equipped with plan achromatic objectives (Leica Microsystems, Wetzlar, Germany). EMD, EFF/L, EFT, EFD, MEMA, and SMCNL were graded semi-quantitatively in HE, αSMA, RF, and MOVAT stained sections in the aortic media. All features were indexed as 0 (none), 2 (mild), 4 (moderate), or 6 (severe) on three predetermined locations (left, middle, and right) of every section, which we refer to as “microscopic fields” maintained in evaluation of all stainings on sister sections. Expression of the VSMC marker αSMA in the intimal layer was graded as being present or not. The elastic lamellae were counted manually in the RF stained sections by two independent observers. 

### 2.4. Statistical Analysis 

All numerical data are presented as mean ±SD of three microscopic fields on each stained slide. For comparison between the groups, statistical differences were evaluated with the Mann–Whitney U-test. One, two, and threeway ANCOVA tests were performed to correct for age and gender. Significance was assumed when *p* < 0.05 using SPSS 20.0 software program (SPSS Inc. Chicago, MI, USA). Graphpad software was used to create graphics of statistical analysis. 

## 3. Results

The ascending aortic wall in adults is historically divided in three layers: the internal layer (tunica intima); the middle layer (tunica media); and the outer layer (tunica adventitia). The tunica intima is mostly described consisting of a single layer of endothelial cells lining the aortic lumen on top of a subendothelial layer of loosely organized elastic fibers and VSMCs. The tunica media is known to contain four major elements: VSMCs, elastic fibers arranged in lamellar units, collagen, and the interposed glycosaminoglycans. The outer part of the tunica media also contains vasa vasorum originating in the tunica adventitia. The tunica adventitia predominantly consists of loose fibrous tissue containing collagen, nerve fibers, fibroblasts, adipocytes, and vasa vasorum lined by endothelium and VSMCs. 

As this study focuses on the developmental stages of the wall of the ascending aorta, we decided not to adhere solely to the already described vascular wall architecture, but in addition, we described the different elements in upclimbing stages for the normal developing vascular wall in TAV compared to age-matched BAV patients. 

### 3.1. Development of the Innermost Layer of the Ascending Aorta

**Figure 1:**
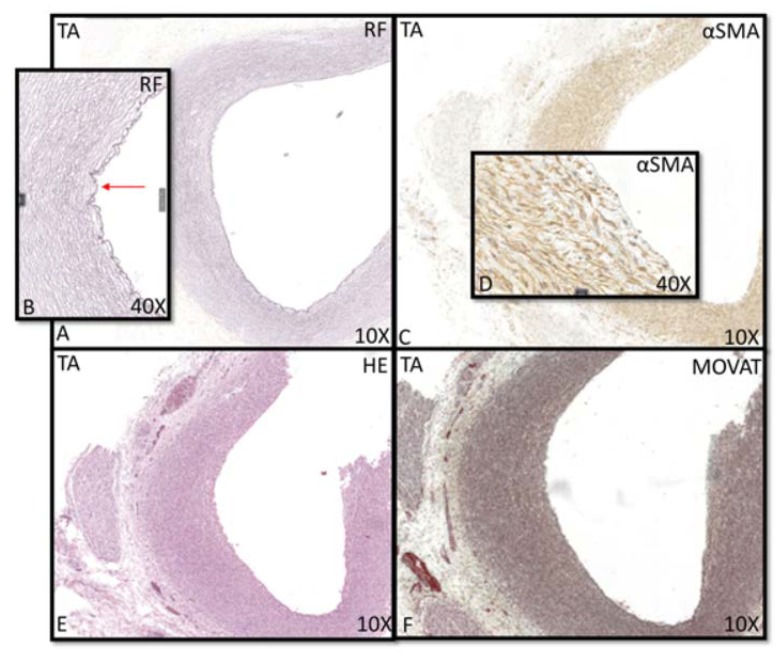
Transverse histological sections of the aortic wall in the non-dilated TAV of 21.5 weeks gestational age (**A–F**) with details (**B,D**). (**B**): arrow pointing towards the lamina elastica interna, being negative in the αSMA staining (**C,D**). In the HE and the RF stained sections (**E,F**), no pathology is seen in the vessel wall. RF: resorcin fuchsin; αSMA: alpha smooth muscle actin; HE: hematoxylin eosin; TA: tricuspid valve without dilation; magnification ((**A**,**C**,**E**,**F**) 10×; (**B**,**D**) 40×).

**Figure 2:**
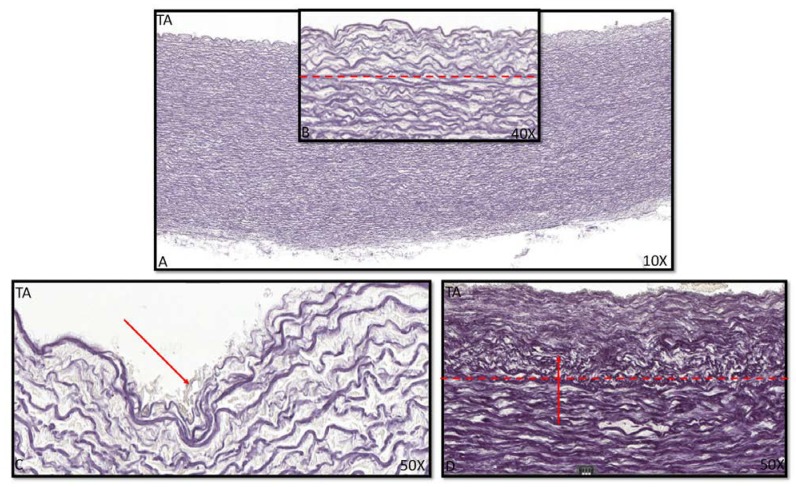
Transverse histological section of the aortic wall in the non-dilated TAV of 4 months old (**A–D**) with details, stained with RF. (**A**): Overview of ascending aortic wall with a detail (**B**) of the intimal layer indicated with the dashed red line. (**C**): The lamina elastica interna fanning out to form an intimal layer in between. (**D**): The dashed line indicates the intimal layer, with the arrow pointing at the fine elastic lamellae in the intima, which appear “thinner” and fragmented as compared to the lamellae in the tunica media. RF: resorcin fuchsin; TA: tricuspid valve without dilation; magnification ((**A**) 10×; (**B**) 40×; (**C**,**D**) 50×).

**Figure 3:**
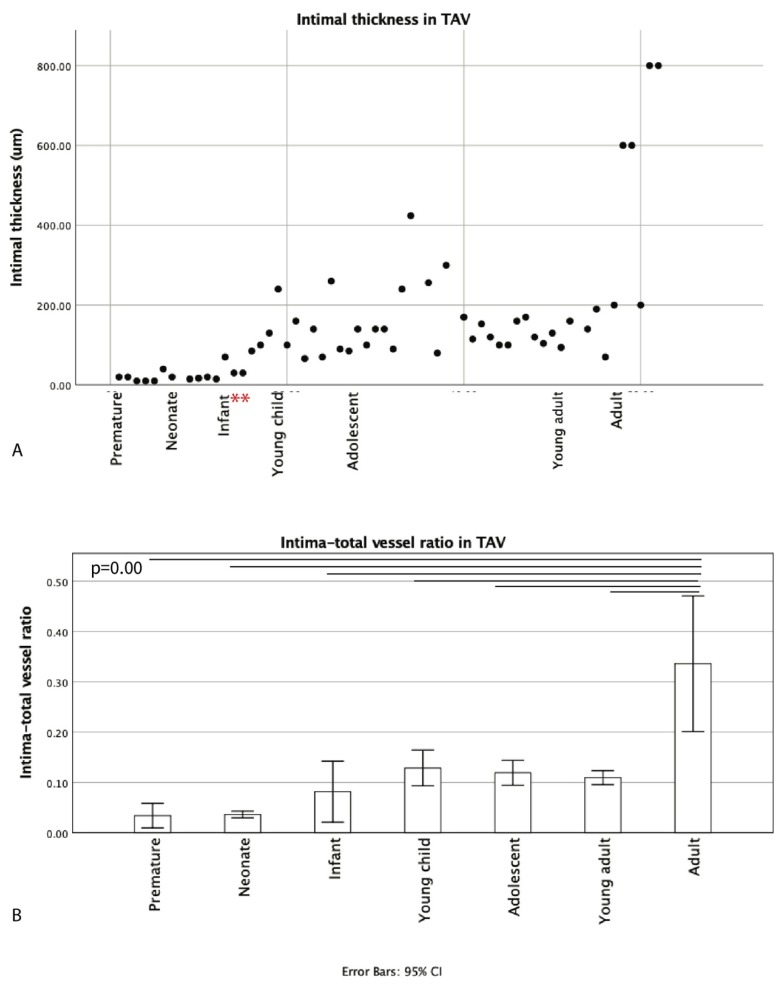
Graph (**A**) showing the intimal thickness (μm). The intimal thickness increases gradually with age, with two outliers at 4 and 6 months (indicated with *). Graph 3 (**B**) shows the ratio between the intima and the total vessel thickness. The adult group significantly had the highest intima–total vessel ratio as compared to all other groups (*p* = 0.00).

The youngest TAV was a 10 weeks GA with an endothelial intimal layer and only showing a thick internal elastic lamina (lamina elastica interna, LEI) as a border with the middle layer of the ascending aorta (Figure 1A,B). This morphology was also seen in the neonatal phase until the age of 1 month (Figure 1). The single layer of the LEI was negative for alpha smooth muscle actin (αSMA) staining (Figure 1C,D). At the age of 4 months, the intima developed a subendothelial layer and started to increase in thickness. The LEI still bordered the tunica media (Figure 2A,B), but locally split (Figure 2C), a process that we describe as fanning out of the LEI. Between the endothelial layer at the luminal surface and the LEI, the subendothelial layer increased gradually in thickness with age in absolute values, with two outliers at the ages of 4 and 6 months (Figure 3A). With increasing thickness, the intimal layer contained fine elastic lamellae, which appeared “thinner” and fragmented as compared to the lamellae in the tunica media (Figure 2D). 

**Figure 4:**
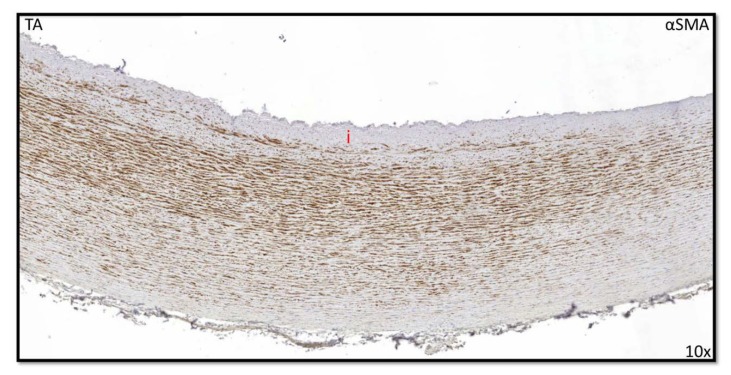
Transverse histological section of the aortic wall in the non-dilated TAV of 2 years old. No expression of αSMA is seen in the tunica intima (i). TA: tricuspid aortic valve without dilation; αSMA: alpha smooth muscle actin; magnification 10×.

However, as the tunica media thickness varied with age (varying from 320 μm to 2000 μm between fetal and adult specimen), as discussed in the next segment, we decided to calculate the ratio of the intima thickness to total vessel thickness rather than comparing absolute diameters (Figure 3B). A trend of increasing intima–total vessel ratio was seen from prematurity until young adulthood, albeit not statistically significant (Figure 3B). The adult group significantly had the highest intima–total vessel ratio as compared to all other groups; however, it was not related to atherosclerosis (*p* = 0.00) (Figure 3B). At two years of age, the intimal layer was still devoid of αSMA (Figure 4). 

**Figure 5:**
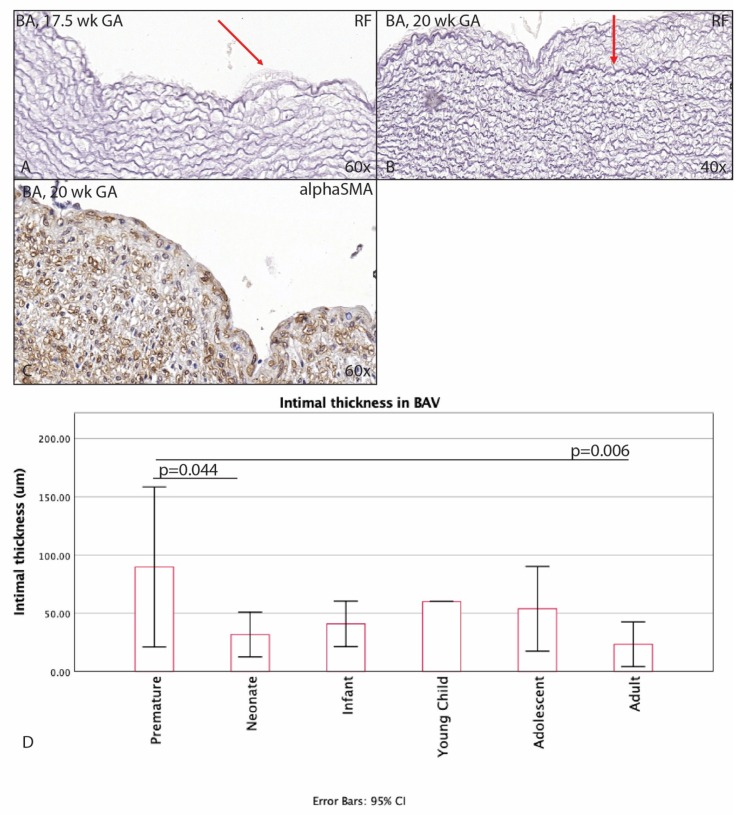
Transverse histological section of the aortic wall in the non-dilated BAV of 17.5 weeks gestational age (**A**) and 20 weeks gestational age (**B,C**). Intimal thickening in BAV is already apparent in the premature phase (**A** and **B** arrow pointing towards tunica intima). The tunica intima is devoid of αSMA expression (**C**). After birth, the intimal layer becomes significantly thinner in the BAV group (*p =* 0.044) and further decreases in adulthood (*p* = 0.006) (Graph **D**). RF: resorcin fuchsin; αSMA: alpha smooth muscle actin; BA: tricuspid valve without dilation; magnification ((**A**,**C**) 60×; (**B**) 40×).

**Figure 6:**
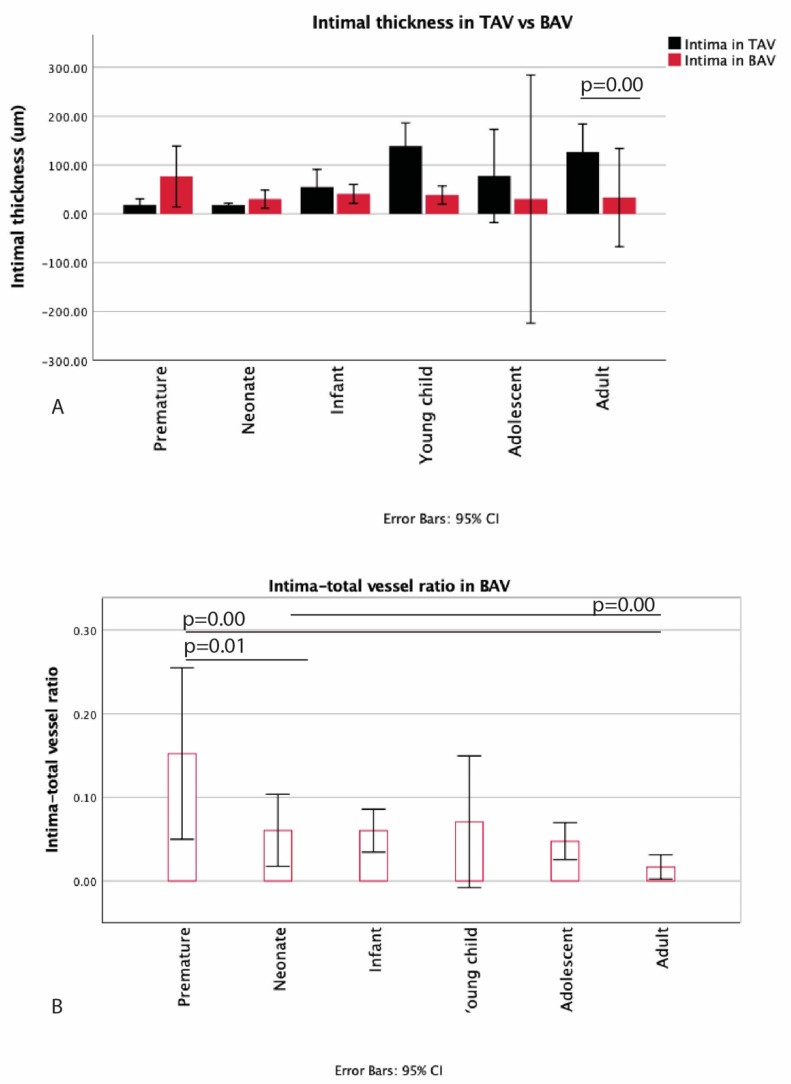
Graph 6 (**A**) compares the intimal thickness between the TAV and the BAV. The difference between absolute thickness in the WHO defined groups is significant between the adult BAV and TAV groups (*p* < 0.01). The intima–total vessel ratio in BAV is seen in Graph 6 (**B**), which reveals a significant decrease after birth (*p* = 0.01), after which, the ratio stabilizes. In the adult, the intima–vessel ratio further decreases and is significantly lower as compared to the premature and the neonate ratio (*p* < 0.01).

The youngest BAV individual was 17.5 weeks + days GA. In contrast to the TAV, this premature BAV already showed some thickening of the subendothelial layer (Figure 5A), increasing further during the premature phase (Figure 5B). This thickened space between the endothelium and the LEI was nearly devoid of αSMA, comparable to the TAVs at this age (Figure 5C). After birth, the intimal layer became significantly thinner in the BAV group (*p* = 0.044) (Figure 5D). In the TAV population, the premature infant remained with a single LEI until the age of 4 months, whereas, in the BAV population, the premature infant showed a thicker layer between the endothelium and the LEI as compared to the premature TAVs. This difference between BAV and TAV was not significant (Figure 6A). After the infant age, the intima in the TAV group increased more in thickness as compared to the BAV group. The difference between absolute thickness in the WHO defined groups was, however, only significant between the adult BAV and TAV groups (*p* < 0.01) (Figure 6A). The MOVAT pentachrome staining did not show any signs of atherosclerosis in the young age groups. 

For the BAV group, we also calculated the intima–total vessel ratio (Figure 6B), which revealed a significant decrease after birth (*p* = 0.01), after which the ratio stabilized. In the adult, the intima–vessel ratio further decreased and was significantly lower as compared to the premature and the neonate ratio (*p* < 0.01). 

**Figure 7:**
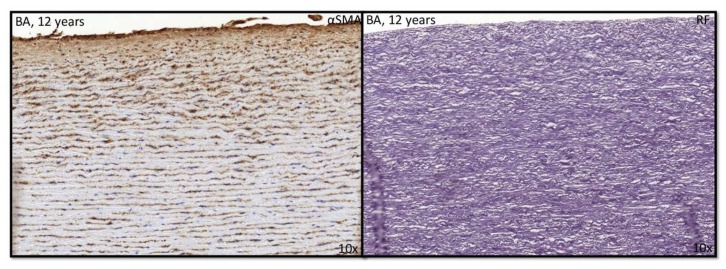
Transverse histological section of the aortic wall in the non-dilated BAV of 12 years. At this age, αSMA expression is seen in the intimal layer. αSMA: alpha smooth muscle actin; RF: resorcin fuchsin; BA: Bicuspid valve without dilation; magnification (10×).

In childhood, the BAV showed αSMA expression in the intimal layer (Figure 7).

### 3.2. Development of the Middle Layer of the Ascending Aorta

The middle layer or media was defined as the area between the LEI and the outermost layer, the adventitia. The media consisted mainly of elastic lamellae, VSMCs, extracellular matrix, and vasa vasorum being confined to its outer part. 

**Figure 8:**
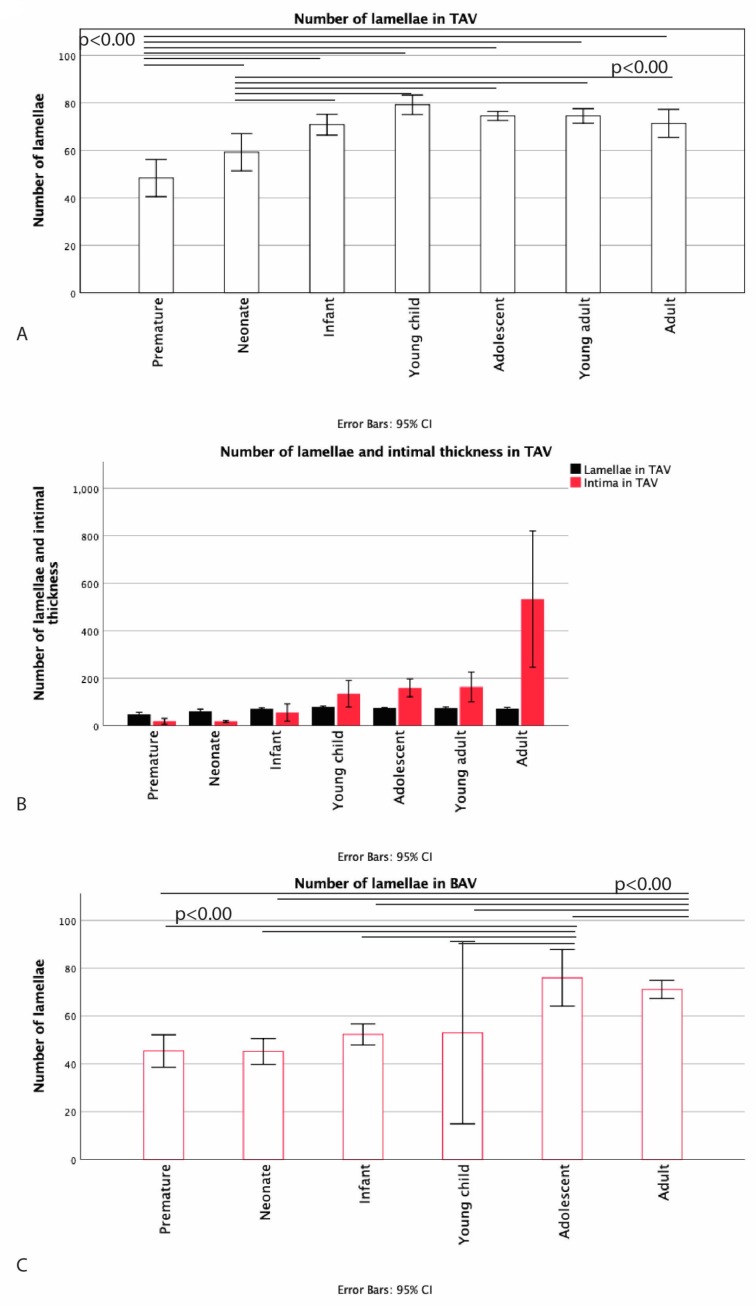
Graph (**A**) shows the number of lamellae in TAV. A significant increase is seen from prematurity up to and including the young child phase, after which the number of lamellae decreases significantly as compared to the young child phase. Graph (**B**) shows the intimal thickness and the number of lamellae in the TAV group. It is apparent that the intimal layer remains relatively thin until the complete lamellae are formed. After the number of lamellae is stabilized, the intimal thickness increases. Graph (**C**) shows the number of elastic lamellae in the BAV. The first significant increase in number of lamellae is seen between the young child and the adolescent phases (*p* < 0.01). The number of lamellae in the adolescent and the adult groups is significantly larger than the rest of the groups (*p* < 0.01).

In the premature TAV, the medial layer consisted of neatly organized elastic lamellae without elastic fiber thinning, fragmentation, or degeneration and with minimal MEMA. Figure 8A shows the number of lamellae in the various groups in TAV with a significant increase between the premature and the neonate groups (*p* = 0.012) between the neonate and the infant groups (*p* < 0.01) and between the infant and the young child groups (*p* < 0.01). The highest number of lamellae was found in the young child group, after which the number remained stable during childhood. The adolescent group showed a slight decrease in the number of lamellae as compared to the young child group (*p* = 0.049), and the adult group also showed a decrease in number of lamellae as compared to the young child group (*p* = 0.018). It was apparent that the intimal layer lacking α-actin remained relatively thin until the complete number of lamellae was formed. After the number of lamellae was stabilized, we found that the intimal thickness increased; this was, however, not statistically significant (Figure 8B).

The number of lamellae in the BAV group in contrast to the TAV group did not increase significantly in the early years of life. The first increase was seen between the young child and the child phases after 4 years of age in our population. Subsequently, the number of lamellae remained stable, which did not decrease in adulthood, as in the TAV population (Figure 8C). 

**Figure 9:**
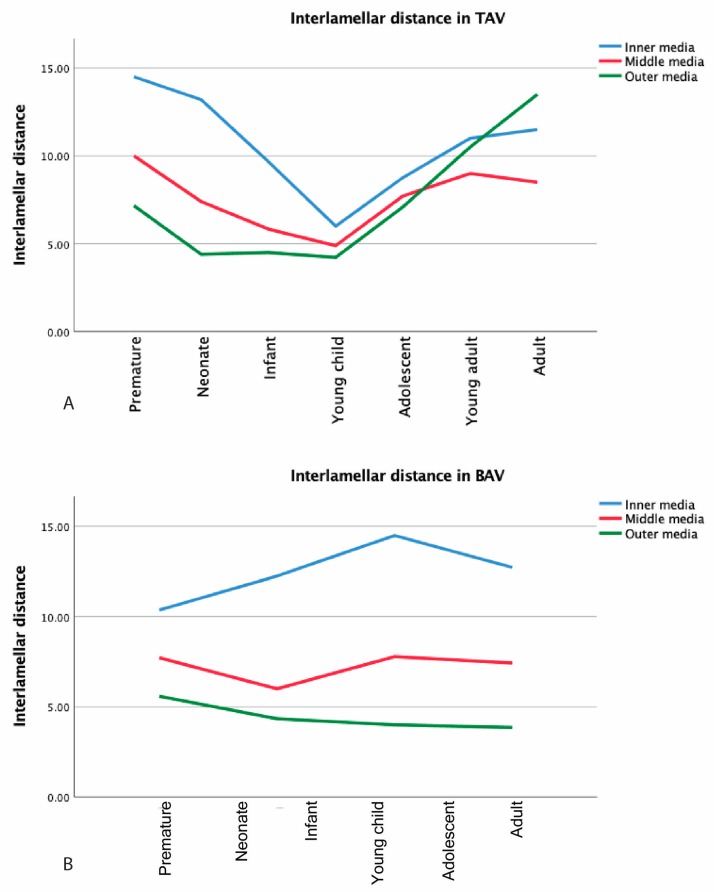
The interlamellar distances are shown for tricuspid aortic valve (TAV) (graph **A**) and bicuspid aortic valve (BAV) (graph **B**) for inner media (IM), middle media (MM), and outer media (OM).

The media could be subdivided in three parts—inner, middle, and outer media—on the basis of the appearance of the elastic lamellae. The interlamellar distance was greatest in the inner media, followed by the middle media, while the outer media showed marked compaction of the elastic lamellae. In the TAV group, the interlamellar distance varied between the groups. Throughout the premature phase until the young childhood, the interlamellar distance decreased within the inner, the middle, and the outer media, while the difference between the three layers remained apparent. From the young child phase, the interlamellar distance increased again but now proportionately in the inner, the middle, and the outer media (Figure 9A). For the BAV group, the distance remained stable throughout the years (Figure 9B).

### 3.3. Development of the Outer Most Layer of the Ascending Aorta

The adventitia varied in thickness in all age categories, which was dependent on the area where the section was taken in all age categories. In all groups, this layer was mainly populated by loose fibrous tissue, large nerve fibers, vasa vasorum, and adipocytes. Over the years, a decrease in size and number of the nerve fibers and an increase in number of adipocytes were seen for both BAV and TAV.

## 4. Discussion

In recent years, literature about ascending aortic wall pathology has increased exponentially. Despite enormous efforts and great progress in research on mechanisms underlying aortopathy, we still do not know exactly what causes aortic dilation and rupture in a certain patient population. A very specific group of patients with an increased risk of aortopathy consists of patients with a BAV, with a population incidence of 1–2%. This congenital anomaly forms the most common heart defect leading to aortic dilation in 50–70% of affected individuals [26]. Most studies describing histology in aortopathy patients have, until now, compared the aorta with human adult ascending aortic wall specimens of a TAV. The pathogenetic mechanism leading to aortopathy can, however, only be understood when the normal vascular developmental processes are known. 

In this paper, we aimed to address the fundamental morphological differences between TAV and BAV patients. We described, for the first time in more detail, an upclimbing age and differentiation range of human ascending aortic wall specimens from fetal age until adulthood and compared the vascular features with an age-matched BAV population. Describing vascular pathology is complex, and most studies lack a standardization in the methods and the description of the vessel wall, which hampers understanding of the complex pathogenesis of BAV aortopathy. As the specimens investigated did not exhibit overt pathology at that young age, the standardized pathology score to describe the vessel wall could not reveal any differences between the studied groups. We did, however, identify an important difference in vascular wall development between the TAV and the BAV, which could explain the increased risk for aortopathy in the latter patient group. 

**Figure 10:**
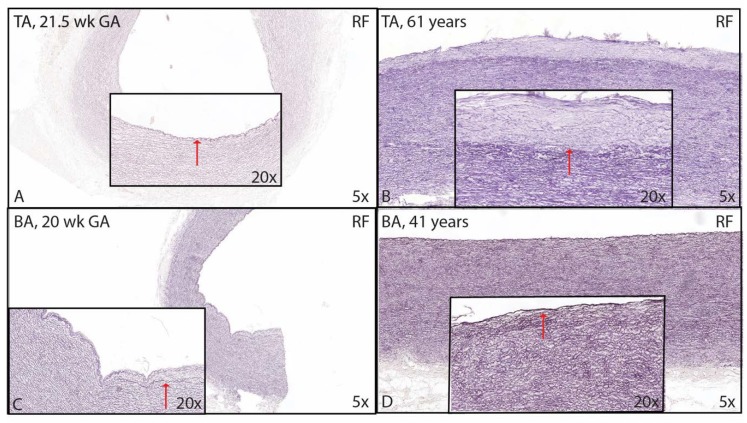
Transverse histological section of the aortic wall in the non-dilated tricuspid aortic valve (TA) (**A,B**) and the non-dilated bicuspid aortic valve (BA) (**C,D**) stained for resorcin fuchsin (RF). In TAV, the intimal layer comprises one internal elastic lamina before birth (indicated with a red arrow in (**A**). The intimal layer increases in thickness during adulthood (border between inner media and intima indicated with a red arrow in (**B**). In BAV, intimal thickening is already apparent in the premature phase (indicated with a red arrow in (**C**). The intima is significantly thinner in the adult BAV ((**D**) arrow pointing towards tunica intima). TA: non-dilated tricuspid aortic valve, BA: non-dilated bicuspid aortic valve; BA: Bicuspid valve without dilation; RF: resorcin fuchsin; magnification ((**A**–**D**) 5× and inserts 20×).

It was previously shown that, in adult BAV, the intima is significantly thinner compared to adult TAV, whereas the media is significantly thicker in BAV [18]. In this study, we confirmed the significant difference in intimal thickness between BAV and TAV adults, although the development of the intimal layer between these two groups was also distinct. In TAV, the intimal development, including formation of a clear subendothelial layer, starts neonatally (4 months). The LEI “fans out”, leading to formation of a layer still bordering the media and a more endothelially oriented layer that continues fanning out. The subendothelial layer increases in thickness with age and also contains the LEI, which continues to fan out; this layer can now be more easily distinguished. Intimal thickness increases absolutely until the young child phase, after which the intimal layer stabilizes for a few years and again increases in thickness during adulthood (Figure 10). This increase in intimal thickness in TAV should be seen as a normal physiological process, which is most probably important for strengthening of the vessel and can be seen as a remarkable ability to adapt to injury and/or regeneration. The intimal proliferation could aid in coping with high and pulsatile blood pressure. When this intimal proliferation process takes on pathological forms, it is referred to as arteriosclerosis [27], and when fatty deposits are included, it is referred to as atherosclerosis [28] with often a deleterious effect on the media. Although the two terms arteriosclerosis and atherosclerosis are often used interchangeably, these are different processes. It was in 1955 that a study group was formed in the World Health Organization in order to delimit atherosclerotic lesions from other alterations (Paterson 1958). They concluded that atherosclerotic lesions are of focal character different from diffuse intimal thickening. According to Jores [21], diffuse intimal thickening develops gradually in the first three decades of life. As the MOVAT pentachrome staining we used did not elucidate that atherosclerosis was present in the young age groups, this supports our hypothesis that intimal proliferation is a physiological process in TAV. How much the mechanism of normal intima formation resembles the development of arteriosclerosis can be debated. In the sense of Jores, it is a compensatory phenomenon in which there is proliferation of the intima and the media and in which there is usually involvement of the arterioles seen in pathological conditions, including pulmonary hypertension [21]. 

It is in this respect of interest that we found a different timing of intimal development and a much thinner intima in BAV, where we know that dilation and even dissection are more common than in TAV [26]. We showed that, in the BAV population, intimal thickening starts much earlier, namely prenatally. After birth, strikingly, the intima decreases in thickness to remain a significantly thinner intimal layer throughout life. A similar thin intima was described in our series of Marfan specimens [23,29]. This could imply that normal protective intimal proliferation is lacking in BAV and MFS, which could increase the risk of aortopathy in these patients. As the current study is descriptive in nature, we need to continue our investigations to further unravel the mechanisms and the genetic pathways involved in formation of the intima. Transforming growth factor beta (TGF-β), for instance, which is known for its fibrotic effects, enhances intimal hyperplasia through the production of extracellular matrix proteins, including the various collagens [30,31]. In a previous study, we already described in BAV that the intima shows a significantly lower expression of TGF-β and its downstream mediator phosphorylated SMAD2 in the intima as compared to TAV [19]. The absence of TGF-β in the BAV intima could therefore explain the lack of intimal hyperplasia [19].

The process of an early intimal development before birth was also described for the development of the ductus arteriosus (DA). In that specific vessel wall, the function of the physiological intimal thickening is important for ductal closure after birth [32]. Splitting of the internal elastic lamina to form the intimal layer, as is seen in our study population, was also described for the development of the ductus arteriosus [32]. 

Postnatally, the medial layer increases in thickness by forming additional elastic lamellae. The number of lamellae is similar between BAV and TAV adults; however, the timing of the development of the lamellae in BAV lags behind that of TAV. Slomp et al. studied DA and aortic tissues and found that, when intimal thickening develops, SMCs in the inner intima migrate, proliferate, and dedifferentiate into the media upon stimuli present during a lasting period of fetal and neonatal stages [33]. A similar finding was found in TAV, as the elastic lamellae in the media only start to form after the intimal proliferation commences. We also found that, initially, the intimal layer is devoid of αSMA expression, suggestive of migratory VSMCs that build the aortic media, whereas, in adults with a fully grown media, the intima layer exhibits αSMA expression. In BAV, however, the intima rather decreases in thickness after birth, as also found in the areas of cytolytic necrosis (CN) in DA. In DA with CN, dedifferentiation and apoptosis of VSMC occur very rapidly (i.e., within a few days), which is not accompanied by proliferation and migration of VSMCs. BAV aortas are characterized by dedifferentiated VSCMs without the ability to undergo a phenotypic switch [18]. Moreover, BAV aortas also exhibit significantly more apoptosis as compared to the TAV [34,35].

Furthermore, the BAV media is significantly thicker compared to the TAV media [18] despite a similar number of lamellae. In the current study, we found that this is attributable to an evident increase in the interlamellar distance. The increase in interlamellar space is caused by, e.g., mucoid extracellular matrix (ECM), which seems to play an important role in the pathogenesis of aortic dilation [36]. The integrity of the aortic extracellular matrix may be compromised by an enhanced activity of matrix metalloproteinases (MMPs), which are crucial in the degradation of ECM proteins. Overactivity of MMPs is possible by several means, including overproduction by VSMCs of the aorta and the inflammatory cells in response to inflammation, oxidative stresses, matrix degradation products, or increased TGFβ-1 activity [19,37,38]. Overexpression of MMPs is a common feature in aortic aneurysms [39,40,41,42,43]. Particularly, MMP-2 and MMP-9 have been implicated in thoracic aortic disease [19,39,40,41,42,43]. We also found an increased MMP-9 activity exclusively in BAV patients susceptible for aortic complications [19]. Recently, it was suggested that a chronic hypoxic environment in the media of aneurysmal ascending aortic specimens could be responsible for the medial degeneration seen in aortopathy [44]. Billaud et al. showed in BAV aortic wall thickening that VSMC hyperplasia and lumen dilation of the vasa vasorum are all changes that could limit nutrient delivery to the tunica media, leading to overt pathology in the vessel wall [44]. They further observed a down-regulated expression of angiogenic and hypoxia-related gene targets in the adventitia associated with evidence of chronic hypoxia in the media in BAV. Interestingly, we found in our previous study that the expression of hypoxia inducible factor 1 alpha (Hif1α) was also increased in the media of only a subset of patients vulnerable for future aortic complications [19]. 

The observed difference in intima and media development in the current study demonstrates why, throughout life, the intima–total vessel ratio decreases in TAV but increases in BAV.

Although the WHO age classification used in this paper comprises eight groups, on the basis of our study, the vascular development can grossly be summarized in three phases for TAV: maturation, stabilization, and degeneration. The maturation phase is characterized by the formation of the elastic lamellae and initial intimal thickening, both seen until the (young) childhood phase. Subsequently, the increase in intimal thickness and the formation of elastic lamellae stabilize, as is also the case for the intima–total vessel ratio. Stabilization lasts until adulthood, after which degeneration sets in with a significant increase in intimal thickening, probably coping with changing physiological conditions. As for BAV, vascular development can be described in two phases taking place in other stadia than in the TAV population: vessel wall maturity and degeneration. Vessel wall maturation takes place prenatally. After birth, the development of the aorta is characterized by aspects of degeneration; the intimal layer does not further increase in thickness, which attenuates the possibility to respond to vascular injury. The VSMCs do not differentiate [18], and hypoxia induces increased degeneration of extracellular matrix, leading to the weakening of the ascending aortic wall and increasing the risk of aortopathy.

**Study limitations:** A study limitation can be considered the descriptive nature of our observations and the relative lack of robustness. However, the access to the upclimbing age series of unique aortic wall material of young human individuals is indispensable for further advance in the field of the study of aortic wall maturation in health and disease. We designed our study by comparing the expression of a panel of markers in the non-dilated TAV and BAV specimens with increasing age. A limitation of our study is that we did not have frozen tissue samples of all the aortic wall specimens we received fixed in formalin from the various groups. Therefore, we could not perform a Western blot to correlate this to our findings of immunohistochemistry. According to the WHO, we subdivided the study population in eight categories. We realize that the number of patients in certain groups is low. However, we still decided to maintain the subgroups in this study to have a complete picture of upclimbing ages but had to combine the young child and the child groups in TAV to make statistical analysis possible. To secure greater confidence in the obtained results as well as determine the underlying mechanisms, an animal model that investigates the maturation of the ascending aorta using knock down or inhibition of the differentiation of vascular smooth muscle cells is needed.

## Figures and Tables

**Table 1:** Gives an overview of the samples included in the study.

Age Categories	Tricuspid Aortic Valve *N* = 60	Bicuspid Aortic Valve *N* = 32
Premature (<38 weeks gestational age)	*N* = 6 10 WK (*n* = 1) 18 WK (*n* =1) 19 WK (*n* = 2) 21.5 WK, D (*n* = 1) 23 WK (*n* = 1)	*N* = 5 17.5 WK, D (*n* = 1) 19 WK (*n* = 1) 20 WK (*n* = 1) 36 WK (*n* = 1) 37.6 WK, D (*n* = 1)
Neonate (0 < 30 days)	*N* = 5 1 D (*n* = 2) 2 D (*n* = 1) 11 D (*n* = 1) 13 D (*n* = 1) 21 D (*n* = 1)	*N* = 6 1 D (*n* = 2) 2 D (*n* = 1) 11 D (*n* = 1) 13 D (*n* = 1) 21 D (*n* = 1)
Infant (1 month < 2 years)	*N* = 6 1 M (*n* = 1) 4 M (*n* = 1) 6 M (*n* = 2) 9 M (*n* = 1) 1.5 Y (*n* = 1)	*N* = 6 1 M (*n* = 1) 3 M (*n* = 2) 4 M (*n* = 1) 5.5 M (*n* = 1) 11 M (*n* = 1)
Young child (2 < 6 years)	*N* = 9 2 Y (*n* = 5) 3 Y (*n* = 2) 4 Y (*n* = 1) 5 Y (*n* = 1)	*N* = 2 2 Y (*n* = 1) 4 Y (*n* = 1)
Child (6 < 12 years)	*N* = 1 6 Y (*n* = 1)	*N* = 0
Adolescent (12 < 18 years)	*N* = 22 12 Y (*n* = 3) 13 Y (*n* = 1) 14 Y (*n* = 3) 15 Y (*n* = 5) 16 Y (*n* = 3) 17 Y (*n* = 7)	*N* = 4 12 Y (*n* = 2) 13 Y (*n* = 1) 15 Y (*n* = 1)
Young adult (18 < 21 years)	*N* = 5 18 Y (*n* = 5)	*N* = 0
Adult (> 21 years)	*N* = 6 47 Y (*n* = 1) 56 Y (*n* = 1) 59 Y (*n* = 1) 61 Y (*n* = 1) 70 Y (*n* = 1) 70 Y (*n* = 1)	*N* = 9 41 Y (*n* = 1) 42 Y (*n* = 1) 47 Y (*n* = 1) 50 Y (*n* = 1) 56 Y (*n* = 1) 62 Y (*n* = 1) 65 Y (*n* = 1) 70 Y (*n* = 1) 72 Y (*n* = 1)

Overview of all specimens included in the study. WHO= World Health Organization; BAV = bicuspid aortic valve; TAV = tricuspid aortic valve; WK,D = weeks, days gestational age, M = month, Y = year.
